# Current knowledge of the immune reconstitution inflammatory syndrome in Whipple disease: a review

**DOI:** 10.3389/fimmu.2023.1265414

**Published:** 2023-10-13

**Authors:** Xiangyi Song, Ruifeng Duan, Liwei Duan, Lijuan Wei

**Affiliations:** Department of Gastroenterology and Digestive Endoscopy Center, The Second Hospital of Jilin University, Chang Chun, Jilin, China

**Keywords:** immune reconstitution inflammatory syndrome, Whipple disease, intestinal barrier, microbial translocation, regulatory cells

## Abstract

Immune reconstitution inflammatory syndrome (IRIS) is characterized by exaggerated and dysregulated inflammatory responses that occur as a result of reconstitution of adaptive or innate immunity. A wide range of microorganisms have been found to be associated with IRIS, such as human immunodeficiency virus (HIV), *Mycobacterium* and actinobacteria. Whipple disease (WD) is an infectious disorder caused by the Gram-positive bacterium *Tropheryma whipplei (T. whipplei)* and IRIS also serves as a complication during its treament. Although many of these pathological mechanisms are shared with related inflammatory disorders, IRIS in WD exhibits distinct features and is poorly described in the medical literature. Novel investigations of the intestinal mucosal immune system have provided new insights into the pathogenesis of IRIS, elucidating the interplay between systemic and local immune responses. These insights may be used to identify monitoring tools for disease prevention and to develop treatment strategies. Therefore, this review synthesizes these new concepts in WD IRIS to approach the feasibility of manipulating host immunity and immune reconstitution of inflammatory syndromes from a newer, more comprehensive perspective and study hypothetical options for the management of WD IRIS.

## 1 Introduction

Whipple disease (WD) is a rare chronic disorder, which is caused by systemic infection with *Tropheryma whipplei (T. whipplei)* (1, 2). Studies have revealed that both the innate and adaptive mechanisms of the immune response are affected during WD, and immunosuppression, resulting in low CD4+ levels, has been identified as a risk factor for immune reconstitution inflammatory syndrome (IRIS), a phenomenon of acute immune-mediated pathology associated with the rapid reversal of immunosuppression, which has been described in association with HIV infection (3). In WD IRIS, non-specific T-helper 1(Th1) reconstitution and the outbreak of its response cytokines have been clearly delineated, and a consensus regarding the pathological mechanisms has been reached (4). Recent research demonstrated that the increased and synergistic action of tumor necrosis factor-a (TNF-a) and interferon-γ (IFN-γ) causes changes in epithelial apoptosis and tight junction (TJ) proteins which maintain intestinal mucosal epithelial barrier function, and the above changes can be measured using serum markers of microbial translocation (MT) (5). Clinical symptoms range from fever to death (4). Corticosteroids may be effective for some symptoms and currently recommended as a first-line treatment, thalidomide is more effective than corticosteroids in managing the early exaggerated immune response in IRIS (6, 7). It is important to identify the mechanisms underlying the progression of WD IRIS in order to provide a theoretical basis for the timely detection and limitation or avoidance of immune reconstitution. This review examines the pathogenesis and clinical presentation of WD IRIS and discusses potential strategies for disease monitoring and management.

## 2 Prevalence and case definitions of WD

WD is a rare and multisystemic chronic infectious disease with an estimated prevalence ranging 3/1000000 in Italy to 9.8/1000000 in the United States (8, 9). Previous literature also describes an incidence rate of 1 in a million and a susceptibility of middle-aged white males (10). A large sample size in the United States study showed that: it affects males and females at similar rates and is more common in Caucasians, non-Hispanics, and people > 65 of age (9). This difference is likely due to sample size and diversity in case series design. HLA-DRB1*13 and DQB1*06 class II alleles were significantly more common in patients with WD. In particular, HLA-DRB1*13, DQB1*06, and DRB1*15 are associated with classic WD (CWD) (11). CWD is the most commonly diagnosed form of WD, with an incidence of less than 1 in a million. CWD primarily affects the gastrointestinal tract, causing primary symptoms of diarrhea in approximately 70-80% of cases (12–14). Some in-depth studies on the intestinal barrier of WD complicated with IRIS are based on this type, which will be introduced in detail in the following. Localized WD (LWD) is one or more extraintestinal organs in the absence of the gastrointestinal tract, predominantly endocarditis and encephalitis. Furthermore, the extraenteric WD variants with cardiac and neurologic spread of infection are characterized by HLA-B*51 and B*44 class I alleles (11). The acute infection is considered to be the first contact with the bacterium. It has been observed in gastroenteritis, fever, or pneumonia mainly in children. Because of its self-limiting or transient nature, it is also called “transient WD” (TWD). Asymptomatic WD (AWD) has been described to occur in healthy carriers. The prevalence of AWD varies with geographic location, ranging from 2% to 4% in Europe and up to 75% in Senegal (15–17). Human leukocyte antigen-G (HLA-G), a non-classical HLA molecule with immunotolerogenic activity, has been found to have increased transcripts in the whole blood of patients with T. *whipplei* infection compared to controls and asymptomatic carriers. It may play a role in the pathogenesis of T. *whipplei* infection by mediating immunotolerance (18).

## 3 Definition of IRIS

Immune reconstitution inflammatory syndrome (IRIS) was proposed by Shelburne et al. in 2002 as “a paradoxical deterioration in clinical status attributable to the recovery of the immune system during highly active antiretroviral therapy.” (19) WD can present with a broad range of signs and symptoms, often leading to misdiagnosis (20). The diagnostic criteria for WD require two out of the following three tests to yield positive results: periodic acid-Schiff (PAS) staining, detection of *T. whipplei* by polymerase chain reaction (PCR), or immunohistochemistry. The gold standard diagnostic test is small bowel biopsy, and the classic histological presentation is PAS-positive foamy macrophages within the lamina propria (21). In WD IRIS, because the PCR shows negative results for *T. whipplei* and antibiotics are usually ineffective, re-inflammation is generally a complication of WD rather than a relapse of WD itself (22, 23). A 1.4% mortality rate has been reported (19). Furthermore, when IRIS is a common complication in patients starting antiretroviral therapy, underdiagnosis in resource-limited settings would contribute to high early mortality (24). Because of the wide variation in the clinical presentation, establishing a consensus on the definition of IRIS remains challenging (25).

## 4 Epidemiology and clinical progression of IRIS

A cohort study published in 2010 revealed that IRIS was detected in approximately 10% of individuals with WD while the prevalence of WD IRIS was twice as high in a study conducted at a tertiary referral center for WD in northern Italy (4, 22). However, there was no evidence that the difference in the prevalence of IRIS in patients with WD was associated with ethnicity (4). Risk factors identified for the development of WD IRIS include age between 40 and 60 years, Caucasian ethnicity, and a history of treatment with immunosuppressive therapy for joint pain, which is considered an inflammatory rheumatoid disorder (26). Other significant predictors included a lower baseline CD4+ T-cell percentage (19). WD is often misdiagnosed, with up to 15% of patients with WD presenting with non-specific symptoms; thus, the diagnosis is often missed or significantly delayed (27). WD is often diagnosed at a late stage once obvious signs have appeared (26). For example, weight loss and diarrhea, which typically occur up to 6 years before a definitive diagnosis of WD is made, although this duration is shorter in cases with immunosuppression (28). Determining the onset of IRIS and establishing the course of the disease is complicated by the fact that the symptoms of IRIS may develop gradually as the clinical symptoms of WD slowly disappear during the first weeks of successful antimicrobial treatment. Therefore, there may be an interval when the symptoms of IRIS and WD overlap (4, 29). Not all case reports clearly delineate WD and IRIS, and IRIS is sometimes incorrectly interpreted as an undertreatment of the primary disease or as a relapse (30). The diagnosis of IRIS relies on clinical judgment and requires careful scrutiny of the signs and symptoms over time and a high index of suspicion for this rare complication of WD.

## 5 Pathogenesis of WD and IRIS

### 5.1 WD displays an anergic and hyporesponsive state

Infection with *T. whipplei* results in atypical activation of bone marrow-derived macrophages (BMDMs) with M2 polarization which associated with Th2 response and type I interferon (IFN) response (31). The impaired *T. whipplei*-specific Th1 reactivity also plays an important pathogenetic role (32, 33). The combined effect of the above cause persistent immunological unresponsiveness and cytokine-induced activation of regulatory cells (Tregs) which may be attributed to a peculiar genetic polymorphism in cytokine genes (34).

Macrophage polarization is a dynamic process through which macrophages acquire specific features, including M1 and M2 polarization (35). *T. whipplei*-stimulated macrophages and duodenal tissues acquire phenotypes of M2/alternatively activated macrophages (36) which are linked to the persistence of bacterial pathogens in tissues and the chronic evolution of infectious diseases (37). Live *T. whipplei* also induces a strong type I IFN response, which is essential for bacterial pathogenicity, and induces c-Jun N-terminal kinase (JNK) phosphorylation to promote macrophage apoptosis. The JNK-independent intracellular replication of *T. whipplei* (compatible with M2) is also associated with type I IFN response (31). The crosstalk between type I and type II IFNs affects the host susceptibility to bacterial infection, and α/β IFNs downregulate the expression of IFN-γ receptors (38). As the production of IFN-γ and interleukin-12 (IL-12) is closely related (3), the reduction in IFN-γ may also result from reduced IL-12 production from antigen-presenting cells, which reveal an immature myeloid dendritic cell (M-DC) phenotype, supporting the systemic distribution of *T. whipplei* and perpetuating chronic infection (39) in all relevant tissues. As a result, marked reduction in the local inflammatory response has been reported (40).

The reduced *T. whipplei*-specific Th1 response in patients with WD (32) also leads to reduced production of IFN-γ in both peripheral blood and the lamina propria of the duodenum (36). Imperceptive IFN-γ production is thought to be critical in the pathophysiology of WD (33). In addition to inducing *T. whipplei* clearance by upregulating the small GTPase activity required for phagosome conversion and inducing the formation of phagosomes into phagolysosomes, IFN-γ also suppresses the production of IL-16, which inhibits the fusion of *T. whipplei* phagosomes with lysosomes. This is achieved by regulating the protein level of cathepsin D in macrophages and upregulating the expression of auto- and pro-apoptotic genes and immunomodulatory genes, such as IL-10 and transforming growth factor (TGF-β) (41). In the presence of TGF-β, precursor CD4+ T-cells differentiate towards regulatory cells (Tregs) characterized by the expression of foxhead box P3+ (FOXP3+) which express specific anti-inflammatory cytokines such as IL-10 and TGF-β (42). This is consistent with the immunopathological observations in WD. Patients with CWD have higher peripheral blood Tregs activation and increased duodenal IL-10 and TGF-β secretion (43). IL-16 also induces the recruitment and differentiation of CD4-expressing cells into tolerogenic cells (3). This contribution to local immune tolerance is also enhanced by a membrane or soluble HLA-G by inhibiting TNF expression (18).

### 5.2 IRIS is a rapid reversal of immunosuppression

The more frequently discussed questions about the pathogenesis of IRIS focus on the degree of immunosuppression resulting from the combined effects of the underlying antigenic burden and patient drug history. IRIS is ascribed to the activation of a non-specific Th1 response underlying the lower CD4+ T cell baseline coupled with reconstituted Treg inadequacy (44). The above characteristics can lead to barrier dysfunction, which can be the cause of prolonged microbial translocation (MT). The resulting entry of endotoxin into the bloodstream can lead to immune reconstitution disorders with systemic immune activation in IRIS CWD (5).

In summary, WD induces a combined effect in which the immune system is suppressed, leading to a high antigen load in affected local tissues. Furthermore, up to 50% patients with classic Whipple’s disease are initially misdiagnosed and treated with immunosuppressive drugs, such as disease-modifying antirheumatic drugs (DMARDs), anti-necrosis factor alpha, glucocorticoids, or drugs, with potentially fatal consequences (16). When treatment with these drugs is discontinued, inflammatory signs may rebound (4), and the degree of CD4+ T cells in the peripheral blood reduction increases with the time of immunosuppressant application (44).

Tumor necrosis factor inhibitors (TNFIs) may reduce phagolysosome fusion, resulting in increased intracellular replication and macrophage apoptosis (45, 46). Susceptibility to IRIS depends primarily on the absence of TNF-signaling (47). Moreover, antimicrobials per se can act as proinflammatory stimulants or upregulate Th1 responses (48). It has been shown that IFN-γ production by CD4+ T cells in response to staphylococcal enterotoxin B (SEB) increases significantly in patients with WD developing IRIS during antibiotic treatment but remains low in treated patients without IRIS (44). Antibiotics not only induce dysfunction of the intestinal epithelial tight junction (TJ) barrier but are also associated with dysbiosis of the intestinal microbiota (49).

Crossing of the defective mucosal barrier by gut microbiota was also demonstrated in a study evaluating pathological damage to the small intestinal mucosa in patients with CWD. The study revealed that elevated levels of lipopolysaccharide-binding protein (LBP), lipopolysaccharide (LPS), and sCD14, definitive alternative markers of increased intestinal permeability and MT, provide direct and indirect evidence of this invasion (50). The link between barrier defects in the intestinal mucosa and systemic immune activation in patients with WD developing IRIS has been further investigated and characterized as a storm in TNF-a expression (25), with TNF-a triggering the NF-κB signaling pathway. This pathway is also an index of inflammation and is synergized by the expression of MLCK activated by IFN-γ, an event that is necessary to promote redistribution of tight junction proteins and paracellular permeability (51). However, the disruption of TJs often leads to increased intestinal permeability, a pathological state termed “leaky gut syndrome” (LGS) (52). The leaky intestinal barrier enhances microbial translocation, which can be predicted by markers as described above.

**Figure 1:**
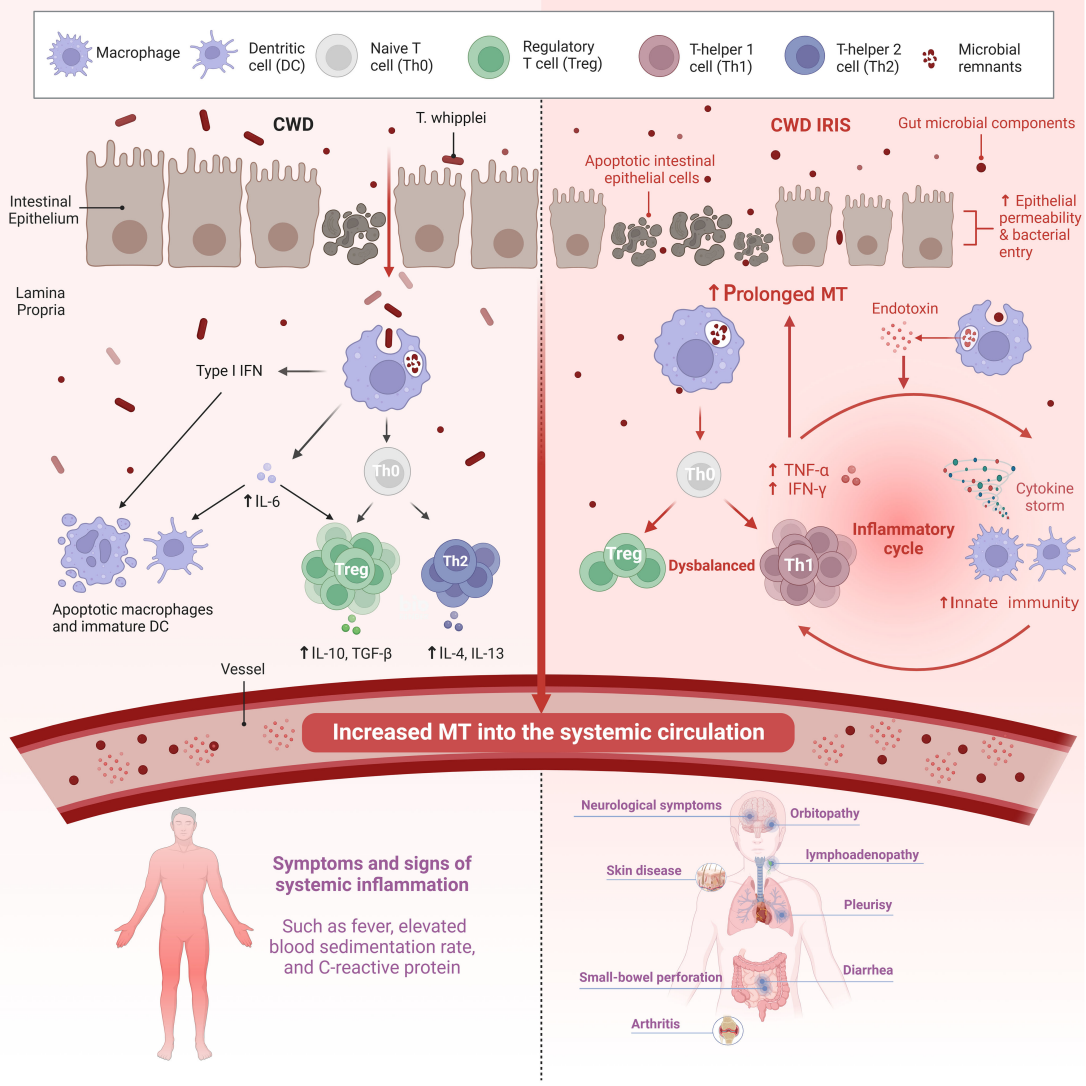
Both the innate and adaptive arms of the immune response are affected in CWD. There is also intestinal epithelial permeability caused by the Th2 cytokine IL-13 and macrophage-derived TNF-a. Increased translocation of gut microbial components across a defective mucosal barrier is an important stimulator of systemic inflammation in CWD. Systemic LPS levels indicate persistent abnormalities in CWD patients independent of active T. *whipplei* infection. In contrast, patients with WD IRIS had more severe damage to the intestinal mucosa, resulting in prolonged MT. Microbial products (in addition to T. *whipplei*) and other bacteria that enter the LP also due to impaired barrier function facilitate intestinal pathology and systemic immune response. However, the mechanism of non-specific T cell reconstitution remains unclear. Although IRIS is an immune overactivation that occurs in the context of a high bacterial load due to immunosuppression, it is driven by non-pathogens. A high systemic antigenic load may trigger inflammation in IRIS-related T cell repopulation, of which an endotoxemic episode may be a potential mediator. Recently, innate immunity has been implicated in IRIS, which may be mediated by endotoxin co-stimulation with non-specific T cell reconstitution. The coupling of innate immunity with adaptive immunity may be responsible for the reconstitution of activated T cells in patients with IRIS. CWD, Classic Whipple disease; IRIS, Immune reconstitution inflammatory syndrome; MT, microbial translocation; IFN, Interferon; TNF-a, Tumor necrosis factor-a; IL, Interleukin; TGF-β, Transforming growth factor. Created with BioRender.com.

Moreover, very recent studies have shown that endotoxaemic episode could be a potential mediator of dysbalanced T cell reconstitution (5). The diverse reservoir of LPS is the gut microbiota, on which LPS depends as a stimulator of the host immune response and as a promoter of pro-inflammatory cytokine secretion (53). The pathologic mechanism leads to an imbalance in T-cell recruitment and inflammatory activation in CWD IRIS, with a positive correlation with sCD14 levels and a negative correlation with the antibody (EndoCAb) titre (5). Therefore, monitoring of inflammatory and microbial translocation markers in patients with WD may be helpful to identify patients who are at risk of developing IRIS and preventing misdiagnosis of treatment failure due to the recurrence of inflammation. However, this intervention needs to be investigated in future studies. **Figure 1** compares the brief process of CWD with CWD IRIS.

## 6 Clinical and pathological manifestations

IRIS in WD usually presents as fever and arthralgia and may progress for several months. The primary pathogenic load area is the duodenal mucosa, with the organs outside of the intestines also posing a concern (44). Besides ocular and skin tissue. IRIS can also affect the lymph nodes, lungs, and central nervous system, as it gives rise to a heterogeneous range of clinical manifestations (57). A common feature when biopsies are obtained from lesions is that local CD4+ T-cell infiltration is typically observed, resulting in varying degrees of tissue destruction. A PCR for *T. whipplei* is usually negative, even if there might be a positive signal from remnants of dead bacteria (12). The reason is that the exaggerated immune response of WD IRIS is driven by pathogen-independent mechanisms (44). There were two articles that counted the clinical manifestations of WD IRIS. The prevalence of IRIS was not statistically different from each other and the overall mortality rate was 10.5% (4, 22).

### 6.1 Fever

Fever is described as a sudden inflammatory response in IRIS in non-HIV patients and can also be caused by an infectious agent (54). Fever is also a typical symptom of WD. Fever that resolves after 48 hours and does not recur is not considered to be IRIS in 80%-86.7% of cases (4, 22). The administration of oral corticosteroids for fever generally shows a response within 24 hours (4).

### 6.2 Arthritis

The incidence of arthritis is high, ranging from almost 93.3% to 100% (4, 22). The investigation of WD-associated arthropathy has been the focus of considerable research. In 1907, Whipple, the pathologist who first described the disease now bearing his name, noted the characteristics of joint involvement which was a part of the clinical manifestations of the first patient with the condition. In 2013, Krol and de Meijer entitled their letter to the editor “Palindromic rheumatism: consider Whipple’s disease.” (27) In 2021, logistic regression analysis was conducted to distinguish WD disease from rheumatoid arthritis, axial spondyloarthritis, psoriatic arthritis, and palindromic arthritis (55). Despite the fact that joint involvement is a general manifestation of WD IRIS, there has been limited research on this subject with some reports in the literature describing the condition as “recurrent arthritis.” (4).

### 6.3 Orbitopathy

Orbitopathy refers to an inflammatory orbital disease with symptoms, including orbital pain, pseudotumors of the eye socket, proptosis, diplopia, and vision loss after hormonal treatment, accounting for 26.7% (4). Unilateral and bilateral orbitopathy has also been reported but is very rare, and biopsy of the superior and lateral rectus extraocular muscles showed CD68-positive macrophages and occasional CD3-positive T cells between the muscle fibers (30). The observed ocular impairment in WD highlights the fact that antibiotics and steroids therapy should not be administered alone but in combination with other measures, such as ocular decompression to prevent ocular damage (56).

### 6.4 Skin disease

Skin disease occurred after WD treatment in 13.3% of cases (4). Subcutaneous erythematous nodules are the main cutaneous manifestations of IRIS in the treatment of WD; these are rare and are most commonly found on the lower limbs, but can also be found on the trunk and wrists (26, 57, 58). Inflammatory reactive dermatoses, which can occur at all stages of WD, including erythema nodosum (EN)-like lesions after antibiotic treatment, are distinct from dystrophic dermatoses, which can be improved with antibiotic treatment but occur only in the late stages of WD. EN-like lesions are a manifestation of high *T. whipplei* burden and have more common clinical, pathological, and immunological features than erythema nodosum leprosum (ENL) (57). IRIS in WD manifests as an ENL. The development of EN-like lesions after antibiotic therapy is also probably caused by lymphangiectasias and immune reconstitution secondary to considerable decrease in the number of viable replicating *T. whipplei* (59).

### 6.5 Small-bowel perforation

Patients with WD primarily affecting the muscular layer of the small-bowel wall may be at a risk of small-bowel perforation once IRIS develops. This incidence is the same as for skin diseases (4). This may be related to increased intestinal permeability caused by increased apoptosis of small intestinal mucosal epithelial cells and shorter villi length (5). This risk cannot be excluded with the use of oral steroid hormones. Evidence indicates that *T. whipplei* mainly infiltrates the submucosa or myenteric layer of the small intestine as PAS-positive material in macrophages containing *T. whipplei* remnants, and PCR for *T. whipplei* performed on biopsies shows negative results; this results in disruption to the integrity of the myenteric layer, and the infiltrate consists mainly of CD4+ T cells with a small number of CD8+ T cells (44).

### 6.6 Diarrhea and associated microscopic manifestations and laboratory tests

Diarrhea was not previously considered a symptom of WD IRIS (4), although some studies list diarrhea as a clinical presentation in patients with WD IRIS (4, 22, 44). Mounting evidence suggests that the reduced duodenal villus length and microbial translocation observed in patients with IRIS affect barrier dysfunction (5, 44) which may contribute to intestinal barrier leakage and gut microbiota and is another vital causative element of autoimmune disorders (60).

#### 6.6.1 Altered epithelial barrier function

Although one hypothesis is that enteric *T. whipplei* does not directly cause diarrhea; rather, it might result from different sanitary and climatic conditions (61), and restrictive structural alignments of the intestinal mucosal barrier were observed. Diarrhea in patients with CWD is associated with the dysfunction and integrity of the intestinal mucosal epithelium (50). Duodenal villus length was shorter in patients with IRIS than in those with non-IRIS CWD both before and after treatment and displayed a lower regenerative potential of the small intestinal mucosa during the course of the disease, as exemplified by an increase in the number of apoptotic epithelial cells, reinforced by a persistently low proliferation rate in the crypt. This situation did not improve even after targeting with *T. whipplei* antimicrobials, whereas villus length was restored in patients with CWD, which seemed to be sufficient to achieve clinical remission (5).

#### 6.6.2 The barrier dysfunction of the intestinal mucosa and systemic immune activation

The role of the inflammatory cytokines that can disrupt barrier function in IRIS was further investigated. TNF-α reduced the percentage of G2/M phase cells and IFN-γ treatment increased the apoptotic rate. Studies in cattle revealed their common role in directly disrupting the intestinal epithelial barrier (62). The intestinal mucosa of patients with CWD who later develop IRIS has a lower number of Tregs, which might involved in local inflammations. The local inflammations disrupts the integrity of the intestinal mucosal barrier and allows the translocation of luminal antigens displacement compared with patients with non-IRIS CWD (44). In a CWD research subject, gut mucosal barrier dysfunction has been shown to contribute to a systemic immune response, as reflected by an increased erythrocyte sedimentation rate, elevated C-reactive protein, and leukocytosis (50). However, the inflammatory activation observed in patients with colitis, which is accompanied by increase in IFN-γ, leads to colonic permeability, suggesting that it is only an early event that contributes to barrier dysfunction but is not sufficient to cause clinical symptoms (51). This may be one of the reasons why extraintestinal disease is prevalent and can present without any gastrointestinal signs (63).

The rates of diarrhea and weight loss differed significantly between different studies. According to the German article, the incidence of both was 33.3%, whereas the incidence in the Italian study was 100% and 80%, respectively. All participants in the Italian study had received immunosuppressive treatment, compared with 80% in the German article. Previous studies have shown that immunosuppressive therapy is associated with the emergence of diarrhea (64). Nevertheless, diarrhea in WD IRIS requires further investigation. Other clinical manifestations include pleurisy, which responds well to oral prednisone, with an incidence of approximately 13.3%. The incidence of lymphoadenopathy disease in the German and Italian studies was 46.7% and 80%, respectively. Neurological symptoms account for nearly 70% to 80% (4, 22).

## 7 Diagnosis of IRIS

The following criteria apply to IRIS in WD: 1) an initial clinical response of symptoms to antimicrobial treatment (cessation of diarrhea and fever, relief of arthritis, and normalization of the C-reactive protein level) within 3 weeks of treatment; 2) recurrence of systemic or local inflammation, with or without fever, lasting more than 1 week, after exclusion of hospital-related conditions; and 3) exclusion of WD recurrence based on histological examination and a negative PCR result for *T. whipplei* despite IRIS manifestation. A diagnosis of IRIS requires that all three criteria are met (4, 22).

PCR does not always differentiate between refractory WD and IRIS at the early stage of treatment, with a short interval between the initiation of antibiotic administration and the emergence of IRIS symptoms (26). There are no published data on the exact time interval for PCR conversion to negative findings following the initiation of antimicrobial therapy, as positive PCR outcomes may also be ascribed to the persistence of inactive pathogens (71) including dead and dying organisms and their residual antigens (12). This highlights the importance of carefully considering the clinical symptoms and signs in order to avoid delays in the management of IRIS.

## 8 When to suspect IRIS in WD

### 8.1 Previous medication regimen and clinical manifestation

As there is no established laboratory test for WD IRIS, the diagnosis is made by clinical observation (65). The immune response to WD IRIS most frequently occurs in patients previously treated with immunosuppressive drugs, such as analgesics, nonsteroidal anti-inflammatory drugs, steroid hormones, and TNFI, for misdiagnosed inflammatory rheumatism (66). Therefore, it is important to be aware of the possibility of IRIS in patients with WD and a history of any of the above medications. It is also important to consider that steroids suppress inflammatory reactions, and for all patients who present with fever or other inflammatory symptoms after the initiation of treatment for WD of no apparent origin, IRIS should be considered, and appropriate management should be instituted (67). In addition, in HIV patients treated with antiretroviral therapy (ART), ENL skin and eye socket pseudotumors that appeared during the first year of antibiotic therapy were all indications of IRIS. Despite treatment, patients with WD may still develop IRIS as the similar underlying pathology is still present. ENL skin is symbolic of a shift to Th1-mediated inflammation (57).

### 8.2 The levels of biomarkers or other laboratory tests

Compromised intestinal immunity with increased microbial translocation into the systemic circulation has been discussed as a mechanism of immune stimulation in IRIS. Intestinal epithelial dysfunction suggests the need for future research to investigate the length and regenerative potential of the small intestinal mucosa and to monitor inflammation and the MT markers discussed above in patients with CWD (5, 50). In CWD IRIS, reduced duodenal villus length and cytokines produced by non-specific Th1-responses lead to mucosal barrier dysfunction and leaky gut syndrome, which can be reflected by serum markers such as LBP, LPS and sCD14. Local immune activation is reflected by elevated levels of IL-6, CCL2, CCL5, CX3CL1 in the duodenal mucosa. In addition to cytokines, the combination of cerebrospinal fluid IFN-γ and TNF-α concentrations provided a predictive model for tuberculous meningitis (TBM)-IRIS, it has guiding significance for diagnosis and treatment (68). This is what WD IRIS is missing. Only when the auxiliary test is more perfect, the diagnostic prediction model is more likely to be established. In summary, further prospective studies are needed to generate robust evidence on WD IRIS.

## 9 Treatment

No trials have provided conclusive evidence on the optimal treatment of WD IRIS. Current management involves the empirical use of glucocorticoids, although these have been reported to be associated with a number of resistant events, and thalidomide, which is currently recommended as the first-line agent (25, 45).

### 9.1 Corticosteroids

The use of corticosteroids as immunosuppressants to treat seronegative arthritis in patients with no clear diagnosis of WD can exacerbate the disease and trigger progression to IRIS (69, 70). When antibiotic therapy fails or the diagnosis of IRIS is clear, oral corticosteroids are generally effective, and several case reports have indicated that oral corticosteroids can alleviate severe symptoms such as fever and subcutaneous nodules and reduce the levels of inflammatory markers such as C-reactive protein (26, 57, 71). However, with the exception of the use of corticosteroids in TB-IRIS (71), current treatment for IRIS is not evidence-based. Moreover, studies on TB-associated IRIS and IRIS associated with HIV infection have generated conflicting evidence regarding therapeutic benefits and appropriate doses of corticosteroids in terms of prevention and mortality rates (72–75). Effective treatment has been reported following the empirical administration of corticosteroids without distinguishing between refractory WD and WD IRIS (30, 56). Corticosteroid treatment for IRIS involves risks and benefits, and it is important to develop more effective and safer drugs (72).

### 9.2 Thalidomide and infliximab

Thalidomide has been successfully used in a case series and has been proposed as the first-line management for IRIS (76, 77) particularly in the presence of ENL reactions (25, 26). Experiments to investigate the mechanisms underlying the effectiveness of thalidomide have not been conducted, and it is speculated to achieve its effects by downregulating TNF-a expression (70). Successful cases of infliximab treatment for IRIS caused by mycobacteria have also been reported (78). However, in all cases, infliximab was used for the treatment of WD before progression to IRIS. This resulted in disease exacerbation with a negative PCR assay result for *T. whipplei*, which does not exclude the fact that infliximab may contribute to progression to IRIS (79, 80).

## 10 Discussion

WD or CWD is characterized by two stages. The first or prodromal stage is marked by protean symptoms, but notably arthralgias/arthritis and fatigue. The second or classic systemic/gastrointestinal stage is marked by diarrhea, abdominal pain, weight loss, and may include other systemic manifestations, such as fever, lymphadenopathy, anemia, skin pigmentation, and bone involvement (14). The two stages may be close, especially in cases on immunosuppressive therapy, in which aggravation occurs on an average of 26 months after immunosuppressive treatment initiation (1, 76).The clinical manifestations of unresolved WD and new-onset IRIS can overlap, and some signs and symptoms are caused by host immune responses rather than by infectious agents (81).

Although the underlying pathological mechanisms of exacerbated T-cell activation in WD IRIS remain unclear, cytokine storm and symptoms of diarrhea have been reported. WD IRIS is characterized by the overproduction of cytokines, and innate and adaptive immune reinforcement of this process enhances the persistence of LPS, sCD14, and LBP. LBP catalyzes the transfer of LPS to the membrane or soluble CD14 (sCD14), leading to NF-κB activation and cytokine release, which, in turn, promote abnormal immune activation. The pathology of MT in WD IRIS has been clearly demonstrated to be similar to IRIS in HIV. Thus, further research is warranted to determine the extent and mechanisms of MT involvement in IRIS, the probability of causing diarrhea in the course of leaky intestine, and the specific process responsible for the massive production of non-specific CD4+ T cells (82).

Regulatory T lymphocytes are essential for maintaining the homeostasis of the immune system, limiting the magnitude of effector responses and allowing the establishment of immunological tolerance (83). Therapeutic strategies aimed at reconstituting the mucosal barrier and controlling exacerbated inflammation may assist in the prevention of IRIS (5). In addition to thymic-derived Treg cells, the intestine is a preferential site for dependent induction of FOXP3+ Treg cells from naive CD4+ T-cell precursors (84). Recently, considerable efficacy has been achieved with immune tolerance therapies in animal and human models, and partial application to TB-IRIS has been reported; however, targeted experiments are required to determine whether these approaches can be replicated in non-specific T-cell-reconstituted WD IRIS (85). Promising results have been obtained in human Treg adoptive transfer therapy and in animal models of autoimmune diseases using biologicals that increase Treg numbers *in vitro*, including IL-2/anti-IL-2 complexes and rapamycin (86). However, the purification and expansion of Tregs remain problematic because of the lack of specific molecular markers, and Tregs employ several independent mechanisms to prevent different pathological immune responses, presenting both opportunities and challenges in the development of new therapies (87).

The body of knowledge regarding WD IRIS remains to be established, and the complete mechanism remains to be elucidated. Local intestinal responses are becoming increasingly important in systemic immune activation. We can focus on the mucosal immune system. For example, the role of gut-associated lymphoid tissue (GALT) and Peyer’s patch in the adaptive immune response; the mechanism by which T. *whipplei* is taken up from the intestinal lumen and whether it involves microfold cells; and the immunomodulatory role of the gut epithelium as an intestinal effector site in addition to the lamina propria. These need to be further investigated in WD IRIS. With respect to diagnosis and monitoring, a detailed analysis measuring the levels of immune response parameters in conjunction with markers of effector cell activation should be performed to more accurately predict the prevalence, severity, and prognosis of WD IRIS.

## Author contributions

LW: Funding acquisition, Writing – review & editing. XS: Visualization, Writing – original draft. RD: Supervision, Writing – review & editing. LD: Writing – review & editing.
